# Spatiotemporal regulation of Rho GTPase signaling during endothelial barrier remodeling

**DOI:** 10.1016/j.cophys.2023.100676

**Published:** 2023-08

**Authors:** Jeffrey MA van der Krogt, Ivanka JE van der Meulen, Jaap D van Buul

**Affiliations:** 1 Department of Ophthalmology, Amsterdam UMC, University of Amsterdam, the Netherlands; 2 Department of Medical Biochemistry, Amsterdam UMC, University of Amsterdam, the Netherlands; 3 Leeuwenhoek Centre for Advanced Microscopy, section Molecular Cytology at Swammerdam Institute for Life Sciences at the University of Amsterdam, Amsterdam, the Netherlands

## Abstract

The vasculature is characterized by a thin cell layer that comprises the inner wall of all blood vessels, the continuous endothelium. Endothelial cells can also be found in the eye’s cornea. And even though cornea and vascular endothelial (VE) cells differ from each other in structure, they both function as barriers and express similar junctional proteins such as the adherens junction VE-cadherin and tight-junction member claudin-5. How these barriers are controlled to maintain the barrier and thereby its integrity is of major interest in the development of potential therapeutic targets. An important target of endothelial barrier remodeling is the actin cytoskeleton, which is centrally coordinated by Rho GTPases that are in turn regulated by Rho-regulatory proteins. In this review, we give a brief overview of how Rho-regulatory proteins themselves are spatiotemporally regulated during the process of endothelial barrier remodeling. Additionally, we propose a roadmap for the comprehensive dissection of the Rho GTPase signaling network in its entirety.

## Highlights

• Rho-regulatory proteins orchestrate spatiotemporal Rho GTPase activity patterns.
• Cell architecture and post-translational modifications guide Rho regulator dynamics.
• Systems analyses enable the identification of individual Rho GTPase signaling units.
• Computational modeling integrates Rho GTPase signaling components into a network.

**Current Opinion in Physiology** 2023, **34**:100676

This review comes from a themed issue on **Endothelium**

Edited by **Jeremy Pearson** and **Paul C Evans**  

For complete overview of the section, please refer to the article collection, “Endothelium”

https://doi.org/10.1016/j.cophys.2023.100676

2468–8673/© 2023 The Author(s). Published by Elsevier Ltd. This is an open access article under the CC BY license (http://creativecommons.org/licenses/by/4.0/).

## Introduction

The endothelium lines the luminal side of blood vessels where it controls the passage of molecules and immune cells into tissues [1]. It consists of a single layer of squamous cells that are connected by endothelial cell–cell junctions to form a selective barrier [2]. Permeability of this endothelial barrier is centrally coordinated by the actin cytoskeleton in such a way that reinforcement of the actin cytoskeleton makes the barrier more restrictive and disruption of the actin cytoskeleton increases barrier permeability [3]. protein from Rho family (Rho) guanosine triphosphat-ases (GTPases) are small molecules that govern modifications to the actin cytoskeleton and hence govern endothelial barrier integrity 4, 5.

With the use of experimental tools such as mutant analyses and GTPase activity pulldown assays, the basic principles of Rho GTPase signaling have been well established. With respect to the endothelium, this led to the general understanding that the Rho GTPases Rac1 and Cdc42 make the endothelial barrier more restrictive through the formation of lamellipodia and filopodia, respectively, whereas RhoA increases barrier permeability by forming contraction-related stress fibers 6, 7. Many lines of evidence however revealed a more complicated picture of Rho GTPase signaling. Instead of having a fixed purpose, Rho GTPases appeared to generate a downstream signaling cascade depending on the sum of protein–protein and protein–lipid interactions [8]. Owing to extensive research on the regulation of Rho GTPase signaling, we now know that Rho GTPase signaling is highly localized and consists of a complex network shaped by Rho GTPase-regulatory proteins (further referred to as Rho regulators) [9]. Yet, our understanding of how exactly these Rho regulators spatiotemporally orchestrate Rho GTPase signaling is still limited.

Dysregulation of Rho GTPase signaling has been linked to a variety of diseases that involve endothelial barrier dysfunction, among which are vascular pathology [10] and cancer metastasis [11]. Therefore, creating a better picture of Rho regulator dynamics during endothelial barrier remodeling might provide new leads for therapeutic opportunities. Owing to the dynamic interplay between Rho GTPases and Rho regulators, this process demands a comprehensive analytical strategy [12]. In this review, we create an overview of the features that shape Rho regulator dynamics during endothelial barrier remodeling and discuss a comprehensive analytical approach that might facilitate in elucidating the entire Rho GTPase signaling network.

## The actin cytoskeleton governs endothelial barrier permeability

**Figure 1:**
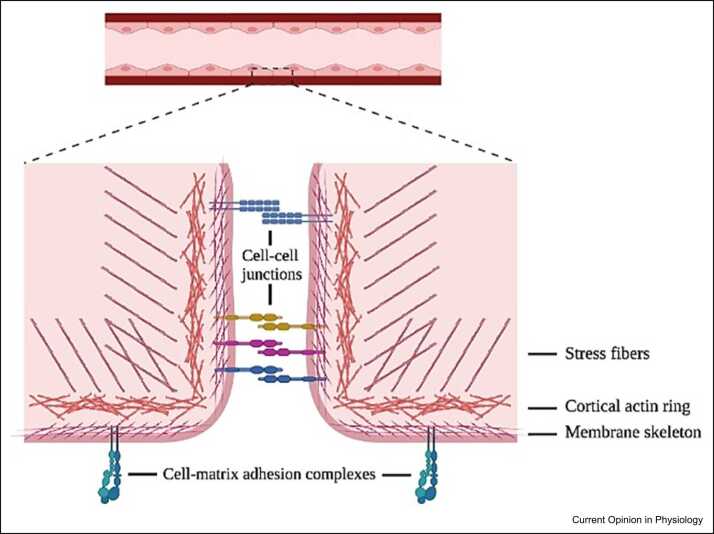
Schematic overview showing the different layers of the actin cytoskeleton in endothelial cells, in relation to cell–cell junctions and cell-matrix adhesion complexes. Created with Biorender.com.

Actin makes up roughly 10% of the total protein in endothelial cells [13]. Based on cellular demand, actin exists either in a monomeric globular form or a polymeric filamentous form (filamentous actin (F-actin)) 14, 15. In its polymerized form, F-actin contributes to the formation of three distinct but interrelated structure components of the actin cytoskeleton (Figure 1). First, located immediately cytosolic to the endothelial plasma membrane is the membrane skeleton [16]. This structure determines plasma membrane shape and facilitates membrane extensibility. Its molecular basis consists of spectrin, which cross-links with F-actin and other binding proteins to form a network that structurally supports the plasma membrane [17]. Second, just beneath the membrane skeleton lies the cortical actin ring. This dense ring interacts strongly with cell–cell junctions and cell-matrix adhesion complexes to generate firm cell adhesions [18]. Third, whereas the membrane skeleton and the cortical actin ring are positioned directly central to the plasma membrane, stress fibers extend throughout the cell cytoplasm. These fibers consist of actomyosin bundles that, upon barrier-disruptive stimuli, contract and so contribute to the formation of endothelial gaps 3, 19.

**Figure 2:**
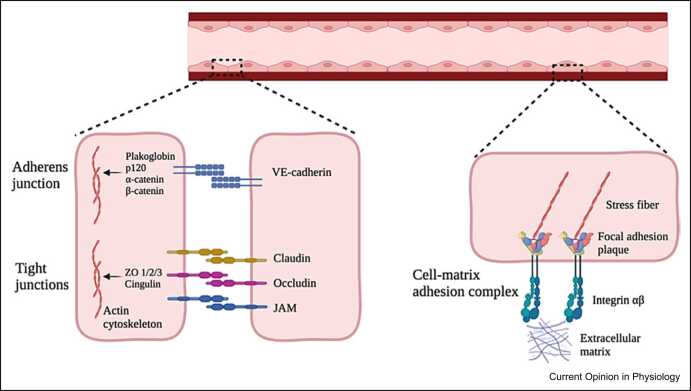
Schematic overview of the direct association between the actin cytoskeleton and adhesion compounds in VE cells, with on the left panel cell–cell junctions and on the right panel a cell-matrix adhesion complex. Created with Biorender.com.

To safeguard a reliable endothelial barrier stability, the cortical actin ring cooperates closely with two types of cellular adhesions, namely cell–cell junctions and cell-matrix adhesion complexes (Figure 2) [20]. Endothelial cell–cell junctions commonly comprise a combination of adherens and tight junctions. Adherens junctions are formed by the homotypic binding between transmembrane vascular endothelial (VE)-cadherin proteins from two neighboring cells [21]. Upon barrier-protective stimuli, the linker proteins plakoglobin, p120, α-catenin, and ß-catenin facilitate the connection between VE-cadherin and the cortical actin ring to increase cell–cell junction stability [22] (Figure 2, left panel, upper junction). Tight junctions arise from homotypic or heterotypic binding between the adhesion molecules claudin [23], occludin [24], and junctional adhesion molecules. Upon barrier-protective stimuli, the linker proteins zona occludens 1/2/3 and cingulin enhance the connection between tight junctions and the cortical actin ring, resulting in a more restrictive endothelial barrier [25] (Figure 2, left panel, lower junctions). Endothelial cell-matrix adhesion complexes are formed by the binding of transmembrane integrin receptors with the extracellular matrix. Within endothelial cells, the interaction between F-actin and the actin-binding proteins vinculin, talin, α-actinin, zyxin, tensin, and filamin leads to the formation of cytoplasmic focal adhesion (FA) plaques [26]. Upon barrier-disruptive stimuli, these FA plaques reorganize in the direction of sites where stress fibers via integrins anchor to the extracellular matrix, where they contribute to the formation of endothelial gaps [27] (Figure 2, right panel).

## Structural Rho GTPase domains manage Rho regulator specificity

**Figure 3:**
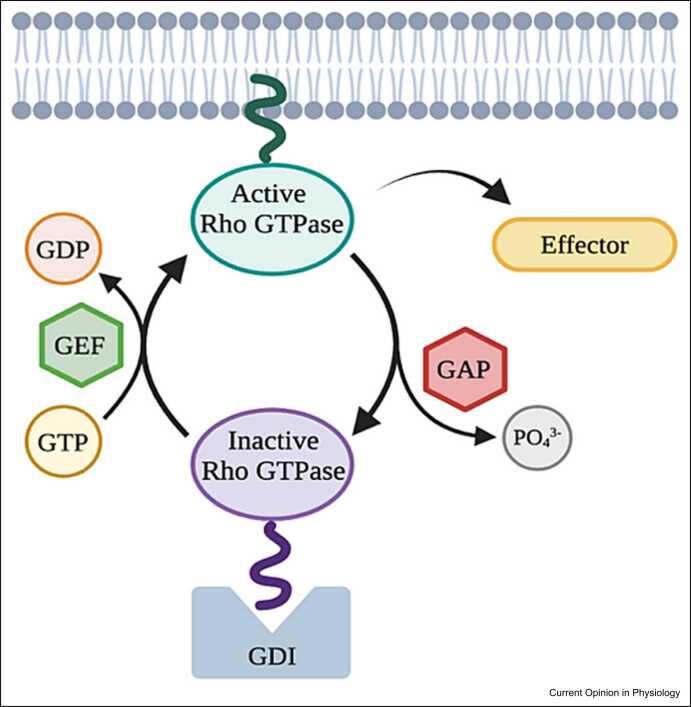
Simplified schematic overview of GTP–GDP cycling. Created with Biorender.com.

The Rho family of GTPases in humans consists of 20 members that can be divided into eight subfamilies [28]. Among these, RhoA, Rac1, and Cdc42 have been most extensively studied. With respect to their mode of activation, Rho GTPases can be considered either classical or atypical. Classical Rho GTPases alternate between an active and inactive state based on whether a guanosine triphosphate (GTP) or guanosine diphosphate (GDP) is bound respectively. This process is formally known as GTP–GDP cycling and takes place under the control of Rho regulators, which include guanine nucleotide exchange factors (GEFs), GTPase-activating proteins (GAPs), and guanine nucleotide dissociation inhibitors (GDIs) (Figure 3) [29].

Rho GEFs activate Rho GTPases by exchanging GDP for GTP [30]. The human genome contains over 80 Rho GEFs that can be subdivided into two families, namely the diffuse B-cell lymphoma (Dbl) family and the dedicator of cytokinesis (DOCK) family. Members of the Dbl family carry a Dbl homology domain, which is oftentimes accompanied by a pleckstrin homology domain [31]. The purpose of the Dbl homology domain is to activate Rho GTPases by catalyzing GDP release. The role of the pleckstrin homology domain is less well defined, but research has shown this domain to fulfill Rho GEF-specific tasks [30]. Whereas in some Rho GEFs, the pleckstrin homology domain contributes to the interaction with Rho GTPases, in others, this domain seems to play a role in autoinhibition [31]. Members of the Rho GEF DOCK family interact with Rho GTPases solely through their DOCK homology domain [32]. In contrast to Rho GEFs, Rho GAPs inactivate Rho GTPases by enhancing GTP hydrolysis. In total, sixty-six mammalian Rho GAPs have been identified [33]. Since, Rho GAPs can act as scaffold molecules, they enable crosstalk between Rho GTPases and other signaling pathways. Rho GDIs, of which three are described in literature, can interfere with signaling events mediated by Rho GTPases. They do this either by inhibiting GDP dissociation from Rho GTPases, inhibiting GTP hydrolysis, or stimulating the release of Rho GTPase from cell membranes into the cytosol [34].

Fundamental to Rho regulator specificity lies the structural core of Rho GTPases, as this determines both the conformation of its binding sites and its subcellular localization. Rho GTPases contain a G domain, a short Rho insert region, and a C-terminal hypervariable region. The G domain is where Rho regulators interact to adjust the activity status of Rho GTPases.

This domain is characterized by five conserved sequence motifs (G1–G5), of which G- domain motifs G2 and G3 resemble the switch-I and switch-II regions, respectively [35]. Consequent to the binding of a Rho regulator, these Rho GTPase switch regions sense whether a GTP or GDP is bound and change their conformation, accordingly, providing a platform for further interaction with upstream regulators and downstream effector proteins. The Rho insert region is located between G-domain motifs G4 and G5. Based on the structural conformation of this region, specific Rho GEFs bind and catalyze the release of GDP [36]. The C-terminus contains a consensus sequence known as the a C-terminal tetrapeptide sequence generally described as having an invariant cysteine (C), two aliphatic amino acids (a1 and a2) and one of several amino acids in the terminal position (X) (CAAX) box and a hypervariable region. The CAAX box carries a lipid anchor that allows the binding of a Rho GTPase to cellular membranes [37]. In a similar way, the hypervariable region is primarily positively charged and thus also engaged with negatively charged phospholipids of cellular membranes. At the plasma membrane, this hypervariable region manages the insertion of lipid anchors into the hydrophobic module of Rho GDIs, resulting in cell membrane release [38]. An overview of how common interactions between Rho GTPases and Rho regulators drive endothelial barrier remodeling in (patho)physiological conditions is provided by Beckers et al. (2010) [39].

## Cell architecture and post-translational modifications underlie Rho regulator dynamics

A general concept that is believed to underlie the spatiotemporal regulation of Rho GTPases relies on the reaction-diffusion system [9]. This system involves successive cycles of (1) local Rho GTPase activation by a Rho GEF, (2) diffusion from a Rho GEF-occupied zone toward a Rho GAP-occupied zone, (3) local inactivation by a Rho GAP, and (4) membrane extraction by a Rho GDI. Seeing that, within this concept, Rho regulators particularly act upon Rho GTPases that reside in proximity, the spatiotemporal regulation of Rho GTPases is highly determined by the distribution pattern of Rho regulators. An important question is therefore how Rho regulators themselves are spatiotemporally orchestrated.

### Distribution of endothelial cell components guides Rho regulator flux

**Figure 4:**
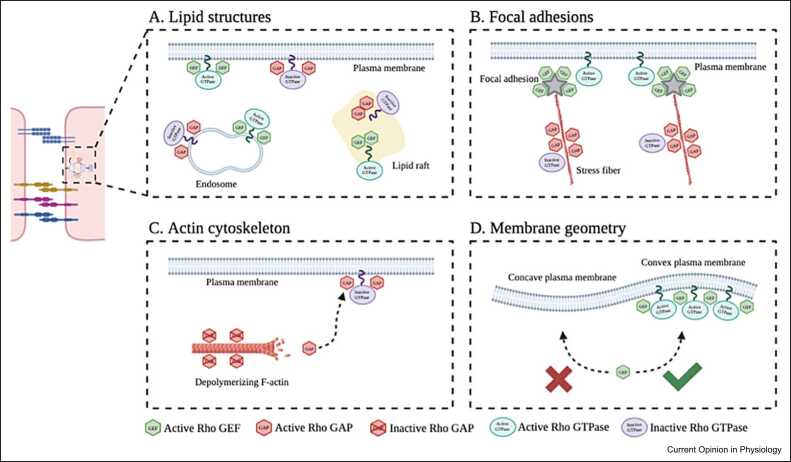
Schematic overview of the regulation of Rho regulator dynamics by endothelial cell components, illustrating regulation by **(a)** lipid structures, **(b)** focal adhesions, **(c)** components of the actin cytoskeleton, and **(d)** plasma membrane geometry. Created with Biorender.com.

One feature that contributes to the intracellular dynamics of Rho regulators during endothelial barrier remodeling is the spatial distribution of cellular components, including lipid structures, FAs, and components of the actin cytoskeleton [9]. First, Rho regulators are known to interact with lipid structures (Figure 4a). This interaction is arranged through the combined attribution of their Dbl homology region domain with either the pleckstrin homology domain or the Bin–Amphiphysin–Rvs (BAR) domain 30, 40. Since these Rho regulator domains differ in their affinity for phospholipids, adjustments in the subcellular lipid distribution directly affect the localization of specific Rho regulators. Indeed, during wound closure, activity zones of RhoA and Cdc42 appeared to portray distinct lipid distribution patterns [41]. Considering receptor tyrosine kinases can adjust the lipid composition of the plasma membrane through the activation of phospholipase C-γ and phosphatidylinositol-3 kinase, they are interesting targets for controlling subcellular dynamics of Rho regulators [42]. Second, Rho regulator dynamics depend on the distribution of FAs (Figure 4b). For example, upon the direct interaction of the Rho GEF ß-Pix with the Rho effector protein p21-activating kinase, cytosolic ß-Pix relocates toward FAs residing at the endothelial cell plasma membrane [43]. Once at the plasma membrane, ß-Pix is phosphorylated by FA kinase, which increases its affinity for Rac1. This leads to the recruitment of Rac1 to the plasma membrane where it is activated by ß-Pix to promote endothelial cell barrier reinforcement [44]. Similarly, upon barrier-protective stimuli, the Rac1 GEFs DOCK180 [45] and Tiam1 [46] specifically bind FA components at the endothelial cell plasma membrane and locally activate Rac1 to reinforce the endothelial barrier. Third, the subcellular localization of actin cytoskeleton components influences Rho regulator dynamics. These components may carry Rho regulators that are being released upon depolymerization of the actin cytoskeleton (Figure 4c). One example includes F-actin, which ties and inactivates the Rac1-specific GAP filamin A (FLNa)-binding RhoGTPase-activating protein (FilGAP). Upon barrier-disruptive stimuli, F-actin is depolymerized, which causes the release of FilGAP. FilGAP subsequently translocates to the plasma membrane where it inhibits Rac1 to facilitate endothelial barrier disruption [47].

In addition to the distribution of cellular components, endothelial cell geometry affects the spatiotemporal dynamics of Rho regulators (Figure 4d). For example, the Fes/Cdc42 interacting protein 4 (CIP4) homology–BAR domain of the RhoA-specific GAP srGAP2 proved to be preferentially engaged with convex membrane curvatures [48]. This raises the idea that srGAP2 can read cell geometry and, through inhibition of RhoA, locally consolidates endothelial cell membrane protrusions [49].

### Post-translational modifications manipulate Rho regulator direction

Although over 150 Rho regulators are involved in the process of endothelial barrier remodeling, these numbers alone do not account for the wide range of actions that 20 members of the Rho GTPase family carry out during this process. In addition to the classical concept of GTP–GDP cycling, other mechanisms contribute to the regulation of Rho regulators to accomplish this plethora of actions [50]. The majority of these relate to post-translational modifications, including phosphorylation, ubiquitylation, and sumoylation. Rho GEFs are oftentimes regulated by phosphorylation, which usually results in their activation either through conformational changes in the catalytic domain for GDP–GTP exchange or by regulating their binding to scaffold proteins that initiate a downstream signal. For example, the stimulation of integrins by an outward mechanical force proved to increase Extracellular signal-regulated kinase (ERK)-mediated phosphorylation of the RhoA-specific GEF-H1. Subsequently, GEF-H1 is recruited to FAs located near stress fibers, where it induces centripetal tension to counteract the outward mechanical forces on the plasma membrane [51]. Beside phosphorylation, Rho GEFs can be targeted for degradation by ubiquitylation. In response to stimulation with hepatocyte growth factor, the E3 ubiquitin protein ligase HUWE1 catalyzes the ubiquitylation of Tiam1, a Rho GEF for Rac1, at sites of cell–cell adhesion, resulting in disassembly of cell–cell junctions and an increased permeability of the endothelial cell barrier 44, 52. Like Rho GEFs, the activity of several Rho GAPs can be regulated by phosphorylation, but its relation to endothelial barrier integrity is yet to be explored. For example, phosphorylation of the Rac1-specific GAP FilGAP has been shown to induce translocation of FilGAP from the actin cytoskeleton toward the cytoplasm [53]. Hypothetically, but not yet confirmed, cytosolic FilGAP might locally inactivate Rac1 to withhold this Rho GTPase from contributing to endothelial barrier reinforcement. Interestingly, the evoked response of Rho regulator phosphorylation on endothelial barrier integrity appears to be determined by the protein kinase involved. Namely, whereas phosphorylation of RhoGDIα by protein kinase A induces a barrier-protective response [54], RhoGDIα phosphorylation by p21-activating kinase-1 induces a barrier-disruptive response [55]. Future directions of research should therefore further investigate the mechanism through which protein kinases regulate the response of phosphorylation and how these protein kinases are spatiotemporally regulated.

Beside post-translational modifications, alternative mechanisms that manipulate Rho regulator dynamics include modifications at the level of gene expression [56], post- transcriptional modifications [57], autoinhibition [58], and crosstalk with other regulatory proteins [34]. Moreover, Rho regulators can interact directly with Rho effectors without the intervention of Rho GTPases. For example, the Rac1/Cdc42-specific GEF adenomatous polyposis coli (APC)–Rho guanine nucleotide exchange factor 4 (ASEF) forms a functional complex with the Rac1/Cdc42 target IQGAP to boost the Rac1/Cdc42 response upon interaction [59]. The interplay of these different signaling components further complicates the course through which Rho regulator dynamics determine Rho GTPase responses, underscoring the need for comprehensive analytical methods to map the Rho signaling network in its entirety.

## Comprehensive approaches toward resolving Rho GTPase signaling

### Systematic perturbation strategies characterize Rho regulator substrate specificity

Over the last decades, molecular perturbation strategies have been used to dissect the spatiotemporal regulation of individual Rho GTPase signaling pathways [60]. Within these strategies, different techniques have been applied to specifically abolish or generate activity of a single component of the Rho GTPase signaling cascade to assess its role during cellular processes. Direct readouts with quantitative information on spatiotemporal dynamics can subsequently be obtained with the use of fluorescence resonance energy transfer (FRET)-based biosensors [61]. FRET is a nonradiative transfer of energy between two fluorophores, whereby the excited-state fluorophore serves as the donor and transfers energy to a ground-state acceptor that resides nearby through long-range dipole–dipole interactions [62]. With the use of FRET-based biosensors for RhoA, Rac1, and Cdc42, Müller P.M. et al. (2020) were able to analyze substrate specificities of 45 Rho GEFs and 50 Rho GAPs [63]. Among the Rho GEFs, 35 portrayed high substrate specificity and ten appeared to regulate multiple Rho GTPases, whereas this was the case for 31 and nineteen Rho GAPs, respectively, indicating Rho GAPs to be more promiscuous. In addition, with the use of the same standardized molecular perturbation strategy, this research group discovered ten previously unidentified activities of Rho regulators and revealed various discrepancies with the existing literature [63]. Together, these findings clearly highlight the potency of standardized molecular perturbation protocols in validating and further characterizing individual Rho GTPase signaling components.

### Computational modeling integrates Rho regulator dynamics into a network

**Figure 5:**
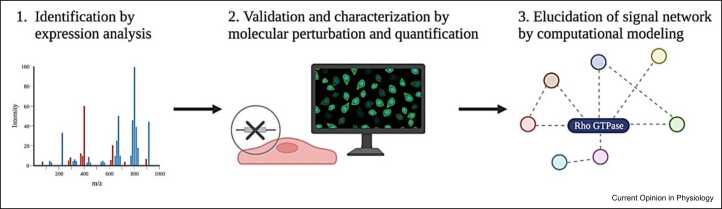
Schematic overview of the proposed roadmap for elucidation of the Rho GTPase signaling network in its entirety, involving (1) identification of individual signal network components by expression analysis, (2) validation and further characterization of signal network components by quantitative readouts of molecular perturbation, and (3) elucidation of the entire Rho GTPase signal network by computational modeling. Created with Biorender.com.

One way to integrate the dynamics of individual Rho GTPase signaling components, as measured by quantitative readouts of molecular perturbation, into a complete network, is by making use of computational modeling (Figure 5). Computational modeling combines the use of mathematics, physics, and computer science to simulate and study complex signaling networks. Through the adjustment of system variables, in this case, the activity of Rho GTPases and/or Rho regulators, computational modeling allows the prediction of experimental outcomes. In general, computational models of cell component dynamics can be divided into four subcategories, namely spatiotemporal, temporal, mechanochemical, and Boolean models [64]. Typically, spatiotemporal models utilize equations based on reaction-diffusion systems. With the use of a spatiotemporal model that incorporated autoactivation, mutual antagonism, and biochemical conservation, Zmurchok and Holmes illustrated how Rho GTPase signaling alone is responsible for reconstructing six out of seven common cell morphologies [65]. This finding revealed that, even in the absence of any intrinsic differences between cells, diverse morphologies may arise from simple adjustments to individual Rho GTPase signaling components.

Boolean models are known for their low computational complexity and their ability to integrate many molecular components, which is beneficial for the simulation of crosstalk with other signaling pathways. For example, Boolean modeling created new insights into the molecular mechanisms involved in endothelial-to-mesenchymal transition (EndMT). Upon drastic changes in tissue requirements, endothelial cells may differentiate into mesenchymal cells. This process inhibits the expression of VE-cadherin and thus leads to loss of cellular junctions, a scenario that is associated with the onset of various diseases. For example, in severe cases of Fuch’s dystrophy, a degenerative disease of the corneal endothelium, EndMT contributes to the development of corneal blindness [66]. Based on a Boolean model, scientists revealed EndMT to rely on an oxygen excess within the extracellular environment as well as on a lack of intracellular VE growth factor-A. This finding illustrates how computational modeling aids in the identification of new therapeutic opportunities for endothelial dysfunction. However, to date, most computational models have been focusing on the better-known Rho GTPases RhoA, Rac1, and Cdc42. To resolve the regulation of Rho GTPase signaling during endothelial remodeling in its entirety, the remaining Rho signaling components need to be recognized for which a systems approach is demanded.

### Systems analysis identifies previously unchartered Rho regulators

In 2020, Müller and colleagues acknowledged the need for a systems analysis to reveal how Rho regulators contextualize and spatiotemporally regulate Rho GTPase signaling [63]. With the use of affinity purification and mass spectrometry, this group laid out a Rho GTPase signaling network consisting of 1292 interactions distributed over 863 proteins. Beside 20 interactions of Rho regulators with Rho effectors and 24 interactions of Rho regulators with small GTPases, 66 interactions were identified between Rho regulators themselves, highlighting a previously unrecognized extensive interplay between Rho regulators. Further characterization experiments identified 34 actin-associated Rho regulators of which only 12 were mentioned in previous literature, and 37 FA-associated Rho regulators of which 31 were not previously affiliated with the integrin adhesion network in the literature [67]. Collectively, the results of this study perfectly demonstrate how a systems-based approach might facilitate the identification of the remaining Rho signaling components.

## Conclusions

Understanding how Rho GTPases orchestrate endothelial barrier remodeling is a long- standing challenge. A central question is how in this course Rho-regulatory proteins are spatiotemporally regulated. Here, we provide an overview of the features that govern Rho regulator dynamics over space and time, including endothelial cell component distribution and post-translational modifications. These features together establish the framework for a model in which Rho GEFs, GAPs, and GDIs contextualize and spatially orchestrate the diffusional flux of Rho GTPases. Moreover, we evaluated comprehensive analytical approaches that may cooperatively map Rho regulator dynamics in the process of Rho GTPase signaling during endothelial barrier remodeling.

A major challenge ahead will be to resolve Rho GTPase signaling in its entirety. Taking into consideration the most recent insights in comprehensive analytical approaches, we propose a roadmap for the dissection of spatiotemporal Rho GTPase signaling networks, including (**1**) the identification of individual Rho GTPase signaling network components with the use of family-wide expression-based systems analyses [63], (**2**) validation and further characterization of Rho GTPase signaling components through quantitative readouts of molecular perturbation experiments [60], and (**3**) integration of individual Rho GTPase signaling component dynamics into a complete cellular network with the use of computational modeling [64].

Ultimately, as Rho signaling is additionally influenced by processes that take place at different physiological scales, future models must consider merging distinct cellular signaling networks into an entire physiological system. Employing higher-dimensional modeling (e.g., three-dimensional models) and multiscale modeling (e.g., mechanochemical models) will likely by inevitable to resolving the exact position of GTPase signaling networks in the development of endothelium-associated diseases.

## Funding

This research did not receive any specific grant from funding agencies in the public, commercial, or not-for-profit sectors.

## CRediT authorship contribution statement

**Jeffrey M.A. van der Krogt**: Conceptualization, Methodology, Formal analysis, Writing – original draft. **Ivanka J.E. van der Meulen**: Conceptualization, Writing – review & editing, Supervision. **Jaap D. van Buul**: Conceptualization, Writing – review & editing, Supervision, Project administration.

## Declaration of Competing Interest

Nothing declared.

## Data Availability

No data were used for the research described in the article.
